# Ovarian and Pelvic Dermoids

**Published:** 1904-02-20

**Authors:** 


### OVARIAN AND PELVIC DERMOIDS.

In a lecture delivered at the Samaritan Free Hospital, Mr. Alban Doran [1] discussed some of the clinical problems connected with ovarian and pelvic dermoids. He pointed out that the great majority of abdominal dermoids are ovarian, and, moreover, that a dermoid tumour found in the abdomen quite separate from the uterine appendages may be of ovarian origin, the tumour having become twisted off its pedicle. In such a case it is important to examine the uterus and its appendages, not only to see if the appendages be wanting on one side, but to ascertain the state of the opposite ovary, for ovarian dermoid disease frequently affects both sides. It is true that an abdominal or pelvic dermoid may be non-ovarian, for the condition has been found in a male, apart altogether from the testicle, but such cases are exceedingly rare, whereas dermoid of the ovary is a common disease. The percentage of dermoids in relation to other ovarian tumours is much higher than was once supposed, for mixed adenomatous and dermoid multilocular cysts are quite frequent.

Simple ovarian dermoids are nearly always pedunculated, a circumstance which is favourable to operation. Ovarian cysts in children are nearly always dermoid, so that when such a tumour is to be operated on the possibility that both ovaries may require removal must not be overlooked, and the consequences of double ovariotomy must be explained to the patient's friends. The condition may be met with at any time of life, in children, adults, and old women.

There are four clinical features of special interest in relation to the ordinary ovarian dermoid. (1) If diagnosed early, ovariotomy proves easy and highly successful. (2) Twisting of the pedicle and worse complications are very frequent. (3) Association of an ovarian cyst with pregnancy, labour, and the puerperium is specially serious when the tumour is dermoid. (4) Neglected dermoids cause dangerous and intractable complications. Early diagnosis of this condition is, therefore, one of the greatest services which a medical man can render to a patient.

The tumour is commonly of small or moderate size, usually occupying the middle of the abdomen up to or above the umbilicus. Free fluid in the peritoneal cavity is very unusual. As a rule fluctuation in the tumour is obscure, and there is a doughy feeling on palpation. Above all, irregular degrees of consistence in different portions are very characteristic of ovarian dermoids. When quite small the cyst not rarely lies altogether in the pelvis, and occasionally remains there even after attaining a considerable size, in which case it is a distinct source of danger if the patient becomes pregnant.

Complications often occur early. Ovarian dermoids are particularly liable to torsion of the pedicle, intracystic hæmorrhage, the formation of adhesions due to inflammation of neighbouring peritoneum, septic infection, and escape of the contents from the cyst cavity, *e.g.*, into bladder or rectum, or, if due to rupture, into the peritoneum. Even very early in its history a dermoid may be tender to palpation, a fact which is an aid to diagnosis. A sudden attack of acute pain is suggestive of a twisted pedicle. The existence of a dermoid tumour in a pregnant woman is a matter of gravity. As a rule gestation is not prejudiced, but complications are liable to arise as pregnancy advances, and during delivery, while the cyst is much exposed to infection during the puerperium. Experience has shown that the removal of a dermoid during pregnancy is not dangerous, whilst abortion does not necessarily follow the procedure, and should it ensue it hardly interferes with recovery from the operation. Hence a pregnant patient who has an ovarian cyst should be strongly advised to have the tumour removed without delay.

[1] Medical Press and Circular, Feb. 10th, 1904.

